# GADD34-deficient mice develop obesity, nonalcoholic fatty liver disease, hepatic carcinoma and insulin resistance

**DOI:** 10.1038/srep13519

**Published:** 2015-08-28

**Authors:** Naomi Nishio, Ken-ichi Isobe

**Affiliations:** 1 Department of Immunology, Nagoya University Graduate School of Medicine, 65 Turumai-cho, Showa-ku, Nagoya, Aichi, 466-8550; 2 Department of Food Science and Nutrition, Nagoya woman’s university, 3-40 Shioji-cho, Mizuho-ku, Nagoya, Aichi, 467-0003 Japan

## Abstract

The prevalence of nonalcoholic fatty liver disease (NAFLD) is increasing in parallel with the prevalence of obesity. DNA damage-inducible protein 34 (GADD34/Ppp1r15a), originally isolated from UV-inducible transcripts in Chinese hamster ovary (CHO) cells, dephosphorylates several kinases that function in important signaling cascades, including dephosphorylation of eIF2α. We examined the effects of GADD34 on natural life span by using GADD34-deficient mice. Here we observed for the first time that with age GADD34-deficient mice become obese, developing fatty liver followed by liver cirrhosis, hepatocellular carcinoma, and insulin resistance. We found that myofibroblasts and immune cells infiltrated the portal veins of aged GADD34-deficient mouse livers. A high-fat diet (HFD) induced a higher level of steatosis in young GADD34-deficient mice compared with WT mice. Differentiation into fat is dependent on insulin signaling. Insulin signaling in young GADD34-deficient mice was higher than that in WT mice, which explained the higher fat differentiation of mouse embryonic fibroblasts (MEFs) observed in GADD34-deficient mice. Through aging or a HFD, insulin signaling in GADD34-deficient liver converted to be down regulated compared with WT mice. We found that a HFD or palmitate treatment converted insulin signaling by up-regulating TNF-α and JNK.

The prevalence of nonalcoholic fatty liver disease (NAFLD) is increasing in parallel with the prevalence of obesity^1^. NAFLD progresses to nonalcoholic steatohepatitis (NASH) featured by exacerbated intrahepatic inflammation, more intense steatosis, and hepatocellular injury. Increased age is associated with prevalence of significant fibrosis in NAFLD/NASH^2^. Compared with Caucasians, African Americans are significantly underrepresented and Hispanic and Asian individuals are overrepresented^3^,^4^. It is estimated that approximately 3–36% of the general American population has NAFLD, with increased risks to 95% in patients with obesity and 70% for patients with T2DM^5^. Of these patients, it has been reported that 20–30% have NASH and that a subgroup will have the increased risk(s) associated with it for cirrhosis and its long-term complications, including liver failure and hepatocellular carcinoma (HCC)^6^.

DNA damage-inducible protein 34 (GADD34/Ppp1r15a) was originally isolated based on UV-inducible transcripts in Chinese hamster ovary (CHO) cells^7^. Two related genes *Ppp1r15a* and *Ppp1r15b*, encoding the protein GADD34 and CReP recruit a catalytic subunit from one of several related protein phosphatase I (PPP1) isoforms to form a holophosphatase complex that can dephosphorylate several kinases that function in an important signaling cascade. Our laboratory and others have shown that GADD34 dephosphorylates the translation initiation factor eIF2α and inhibits the shut-off of protein translation^8^,^9^,^10^,^11^,^12^,^13^. GADD34 with CUE domain-containing 2 proteins has been shown to dephosphorylates IKK, which inhibits NFκB signaling^14^. It has been shown that GADD34 bound to TSC1/2 suppresses mTOR signaling^15^,^16^. GADD34 has been shown to interact with Lyn, a negative regulator of GADD34-induced apoptosis Recently we have shown that GADD34 binds to Lyn and inhibit G-CSF receptor signaling and GADD34 works to inhibit the proliferation and differentiation of hematopoietic stem cells or myeloid precursor cells^17^,^18^.

During the course of observation of GADD34 -deficient mice in aging, we found that GADD34 -deficient mice become more fatty than WT mice by advancing age. Further we found that aged GADD34 -deficient mice showed fatty liver. In this study we provide evidences that GADD34 inhibits age-related obesity and diet-induced obesity (DIO). High fat diet and saturated fatty acids palmitate induces GADD34 expression. GADD34-deficient mice progresses to obesity and NAFLD/NASH followed by hepatic steatosis, liver cirrhosis, hepatoma and insulin resistance Type II diabetes.

## Results

### GADD34 -deficient mice got higher weight and had fatty liver in normal chow diet (ND) by aging

**Figure 1:**
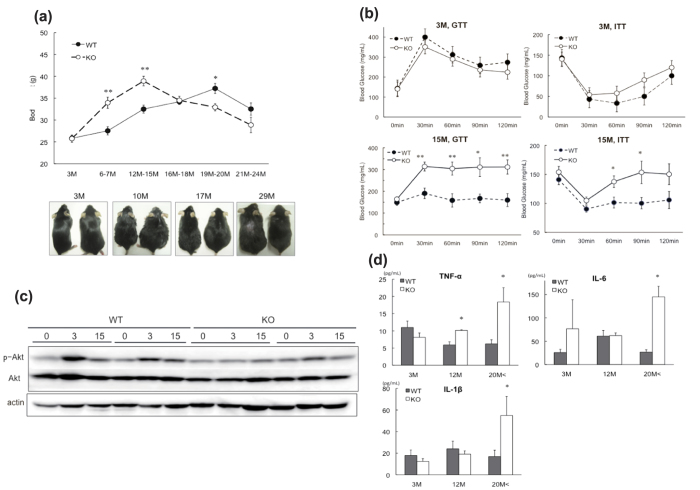
Characterization of GADD34-deficient mice by aging. (**a**) Body weight of male WT and GADD34-deficient male mice (WT; n = 10, KO; n = 10 in each age). Data shown are the mean ratio ± SEM. (*p < 0.05, **p < 0.01). The lower pictures shows 3, 10, 17 or 29 months old WT (left) and GADD34 –deficient (right) male mice. (**b**) GTT and ITT of 3M or 15 M old male WT or GADD34 -deficient mice were examined. Data shown are the mean ratio ± SEM. (n = 4, *p < 0.1, **p < 0.05). (**c**) WT and GADD34 -deficient 17M aged male mice were injected insulin (5 U/mice) to intravenously under anesthesia and liver was remove from these mice in each time (0 min, 3 min, 15 min). Protein expression of p-Akt, Akt and actin in liver were analyzed by western blotting. Data shown are two representative results of four experiments. The original immunoblots are presented in Supplemantary Figure 10a. (**d**) IL-6, TNF-α and IL1-β in serum from 3M, 2M or >20M old WT and GADD34 -deficient mice were measured by ELISA Kit. Data shown are the mean ratio ± SEM. (n = 6, *p < 0.05, **p < 0.01).

Male wild type (WT) and DNA damage-inducible protein 34 (GADD34)-deficient mice were bred on a ND. The body weights of GADD34-deficient male mice were higher until 16–18 months of age, compared with WT mice, after which GADD34-deficient mice lost weight (Fig. 1a). Because obesity is associated with glucose intolerance and insulin resistance^19^,^20^, we examined whether GADD34-deficient mice developed age-related glucose and insulin disorders. With age, male GADD34-deficient mice developed impairments in glucose tolerance and insulin resistance (Fig. 1b). To evaluate insulin signaling, we injected insulin into 17-month-old male WT or GADD34-deficient mice. We found that while the levels of p-Akt (p-Akt2) in liver were increased at 3 min after insulin injection in aged WT mice, they did not increase in aged GADD34-deficient mice (Fig. 1c). We found almost the same results in fat and muscle tissues (Supplemental Fig. 5b). Hepatic Triglyceride (TG) or cholesterol contents in 10–15 months old GADD34-deficient fed ND were higher than those in the same age of WT mice (Supplemental Fig. 3c,d). However, hepatic glycogen contents were not significantly different between 10–15 months old GADD34-deficient and the same age of WT mice (Supplemental Fig. 3b). Insulin resistance might be caused by pro-inflammatory cytokines, and we found that IL-6, TNF-α and IL-1β were up regulated in aged male GADD34-deficient mice (Fig. 1d).

### Aged GADD34-deficient mice had NAFLD/NASH followed by regional fibrosis and hepatoma

**Figure 2:**
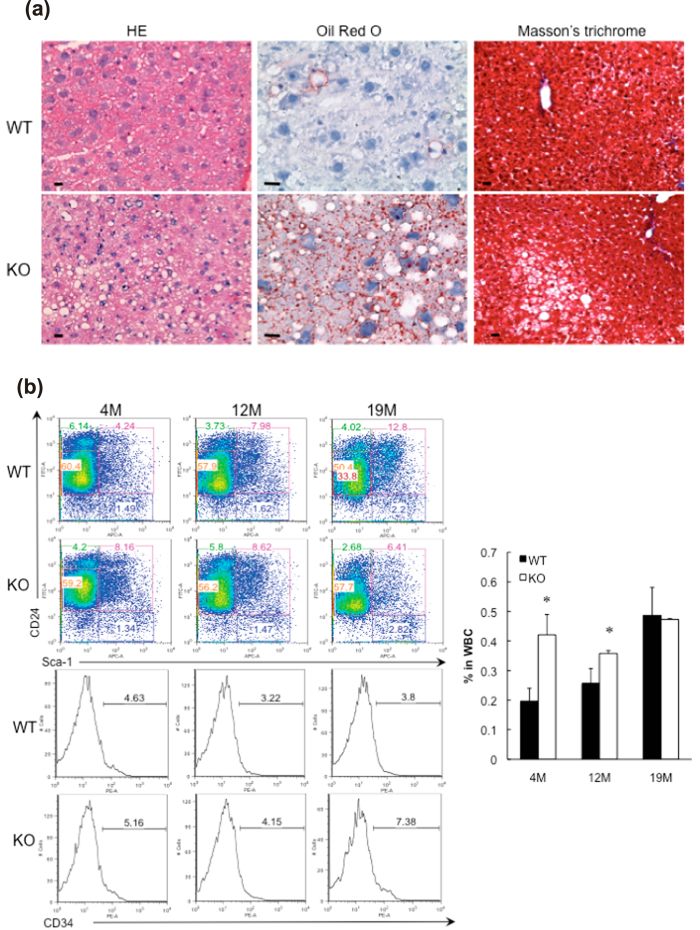
GADD34 -deficient mice accumulated a fat in liver with aging. (**a**) Sections of the liver of 15 M old male WT (upper) and GADD34 -deficient (lower) mice were stained with H&E, Masson’s trichrome and Oil Red O. (**b**) Bone marrow cells were stained with anti-CD24, Sca-1 and CD34 antibodies. Graph (right) shows CD24^+^/Sca-1^+^/CD34^+^ adipocyte progenitor cells in bone marrow. Representative results of three independent experiments are shown. Data shown are the mean ratio ± SEM. (*p < 0.05).

We examined whether GADD34-deficient mice fed ND developed nonalcoholic fatty liver disease (NAFLD)/nonalcoholic steatohepatitis (NASH) with age. Using hematoxylin and eosin (H&E) and Oil Red O staining, we observed higher fat levels in the liver cells of GADD34-deficient mice than in those of WT mice (Fig. 2a). Because bone marrow progenitor (BMP)–derived adipocytes had been shown to accumulate preferentially in visceral fat depots and GADD34-deficient mice showed higher levels of fat droplets and adipocytes than did WT mice with age^21^, we compared these adipocytes between GADD34-deficient and WT mouse bone marrow. GADD34-deficient bone marrow at 4 and 12 months showed higher numbers of CD24^+^/Sca1^+^/CD34^+^ cells than those of WT mice (Fig. 2b).

**Figure 3:**
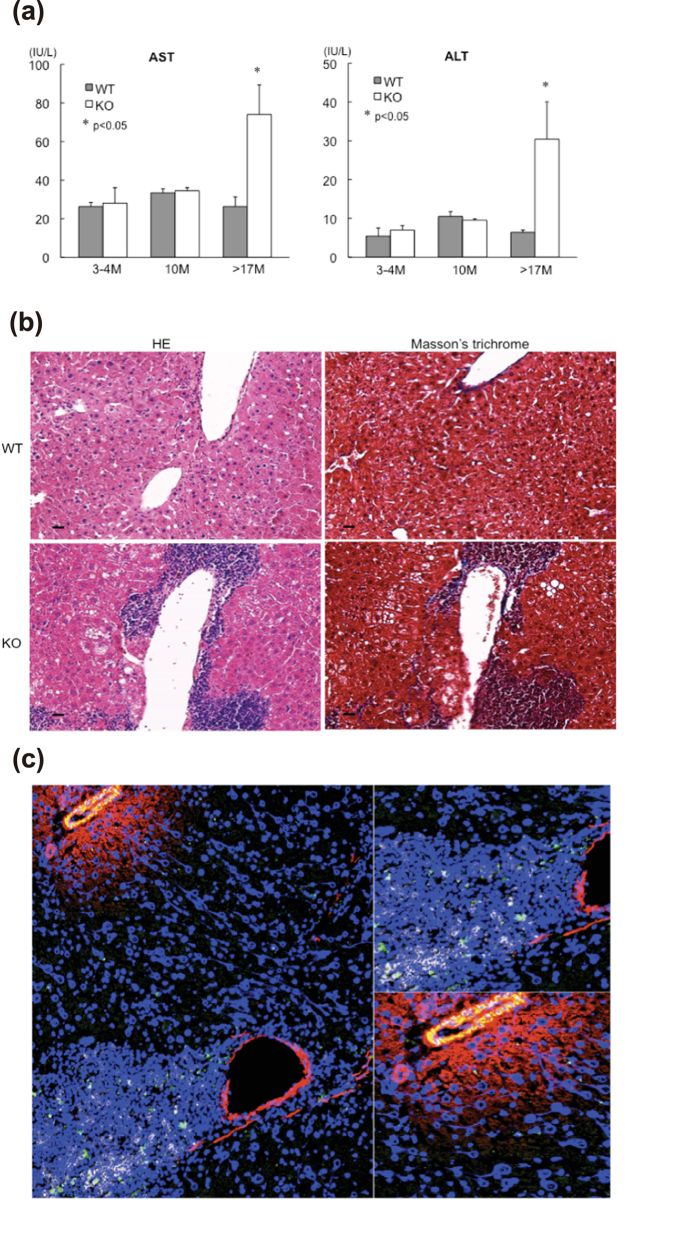
GADD34 -deficient mice developed NASH by aging. (**a**) Concentration of AST and ALT in serum from WT and GADD34-deficient young (3–4M) and aged (10M and >17M) mice were measured by ELISA Kit. Data shown are the mean ratio ± SEM. (n = 6, *p < 0.05, **p < 0.01). (**b**) Sections of the livers of 17 M old male WT (upper) and GADD34 -deficient (lower) mice were stained with H&E and Masson’s Trichrome. Scale bars represent 100 μm. **(c)** Frozen sections of 17 M old male WT (left) and GADD34 -deficient (right) mice liver were stained with anti-α-SMA-PE (Red), anti-GR-1-FITC (Green) and anti-CD4-APC (White).

We found that with age plasma aspartate aminotransferase (AST) and alanine aminotransferase (ALT) levels were up regulated in GADD34-deficient mice compared with WT mice (Fig. 3a). These results suggested that GADD34-deficient mice displayed liver damage, which could proceed to NAFLD/NASH with age. In humans, it has been shown that long-term fatty liver develops into liver cirrhosis. NASH may progress to cirrhosis in 25% of patients and to liver-related death in 10%^22^. We found that some of the aged GADD34-deficient mice began to lose weight, and after autopsy, advanced NASH was apparent, with hard liver and nodules present in some mice. We thought that NASH would proceed to liver fibrosis in GADD34-deficient mice. Fibrosis is defined by the excessive accumulation of fibrous connective tissue composed of extracellular matrix (ECM) components, such as collagen and fibronectin^23^. Collagen staining using Masson’s trichrome showed strong blue intensity in certain parts of the liver of aged male GADD34-deficient mice, whereas age-matched WT mice did not show signs of fibrosis (Fig. 3b). ECM-secreting myofibroblasts are central to the pathogenesis of all fibrotic diseases^24^. We observed myofibroblast migration around portal veins in aged male GADD34-deficient mice by staining for α-SMA, which might induce production of ECM components including collagen. Some immune cell infiltration was also observed in these mice (Fig. 3c). Neither myofibroblast nor immune cell migration was observed in aged male WT liver (Supplemental Fig. 1a). Among aged GADD34-deficient mice, we detected hepatomas by histological examination, with large nuclei and prominent nucleoli and mitosis (Supplemental Fig. 1b). By staining with α-fetoprotein (AFP), we clearly recognized the appearance of HCC (Supplemental Fig. 1c). From these observations, we summarize that male GADD34-deficient mice bred on an ad-lib chow diet gradually accumulated fat, which proceeded to the development of NASH. Advanced liver disease induced cirrhosis or HCC, which proceeded to cachexia with weight loss. The pathological time course of male GADD34-deficient mice with respect to that of WT mice is shown in Supplemental Fig. 2a,b.

### High fat diet induces higher level of steatosis in young GADD34 -deficient mice than that in WT mice

**Figure 4:**
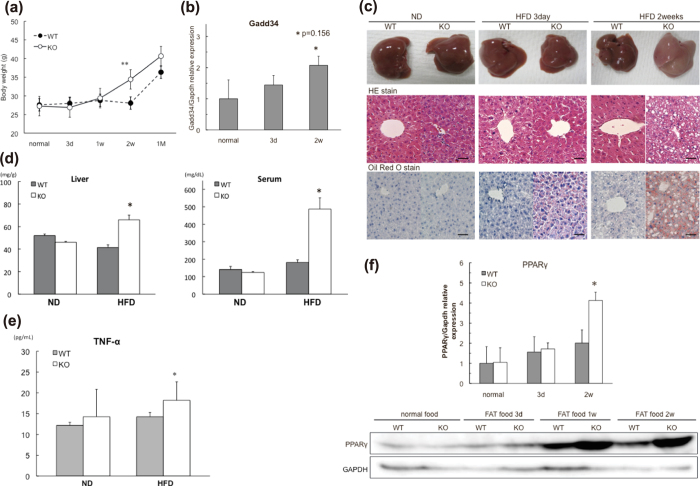
GADD34 deficiency promotes obesity induced by HFD. (**a**) Body weight of WT and GADD34-deficient male mice (WT; n = 6, KO; n = 6) were measured at each time after HFD feeding. Data shown are the mean ratio ± SEM. (*p < 0.05, **p < 0.01). (**b**) GADD34 mRNA expression of the livers of WT mice fed HFD. Data shown are the mean ratio ± SEM. (*p = 0.156). (**c**) Pictures show the representative macroscopic photographs of the livers from WT mice (left) and GADD34-deficient (right) mice fed HFD. Sections were stained with H&E and Oil Red O. Scale bars represent 100 μm. (**d**) Concentration of TG in liver and serum in WT and GADD34-deficient mice fed HFD for 2 weeks. Data shown are the mean ratio ± SEM. (n = 5, *p < 0.05). (**e**) Concentration of TNF-α in serum from 3M old WT and GADD34-deficient mice fed ND (left) or 2 weeks of HFD (right) were analyzed by ELISA. (**f**) PPARγ mRNA expression of the livers of WT and GADD34-deficient mice fed HFD for 2 weeks. Samples were taken at 0, 3 days, one week or two weeks after the start of HFD. Data shown are the mean ratio ± SEM. (*p < 0.05). Protein expressions of the liver were analyzed by western blotting. The original immunoblots are presented in Supplemantary Figure 10b.

The fact that aged GADD34-deficient mice gained weight and developed fatty liver indicates that GADD34 inhibits fat accumulation in the liver. From these findings, we examined whether young GADD34-deficient mice might accumulate fat in the liver due to a high-fat diet (HFD). We bred young (3 months old) male WT and GADD34-deficient mice on a HFD and evaluated body weight, GADD34 expression and liver histology (Fig. 4). We found that the young GADD34-deficient mice were higher in body, liver and epididymal white adipose tissue (WAT) weight compared with WT mice (Fig. 4a, Supplemental Fig. 6a,b). Expression of GADD34 mRNA was up regulated by a HFD (Fig. 4b). Two weeks after starting the HFD, the livers of young male GADD34-deficient mice changed to a whitish color. Histological examination showed that GADD34-deficient mice accumulated higher levels of fat droplets in the liver cells and adipocytes compared with WT mice (Fig. 4c). Fatty liver formation was quicker in young female GADD34-deficient mice than that in male GADD34-defficient mice (data not shown). We observed fat accumulation and inflammatory cell migration in the liver of female GADD34-deficient mice already at 5 days after HFD (Supplemental Fig. 6f,g). The HFD increased triglyceride levels (TG) in liver and serum in young male GADD34-deficient mice, but to a lesser extent in WT mice (Fig. 4d). TNF-α production in serum of aged mice (Fig. 1d) and HFD-fed GADD34–deficient mice was higher than that in WT mice (Fig. 4e). PPARγ, which is the main transcription factor involved in lipogenesis^25^,^26^, was highly up regulated by the HFD in GADD34-deficient mice (Fig. 4f).

It has been shown that a HFD induces myeloid-lineage cell infiltration into fat tissues^27^,^28^. We examined the migration of myeloid-lineage cells by FACS analysis in epididymal WAT of both male WT and GADD34-deficient mice. The HFD increased the Gr-1^+^CD11b^+^ cell population in both WT and GADD34-deficient mice, and the Gr-1^high^CD11b^high^ population of neutrophils was higher in both normal diet- and HFD-fed GADD34-deficient mice. The F4/80^high^Ly6G^int^ population of macrophages was highly increased in GADD34 -deficient mice fed the HFD. The dendritic CD11c^high^Ly6C^int^ cell population was also highly increased in the HFD-fed GADD34-deficient mice compared with WT mice (Supplemental Fig. 6c–e).

### Mechansm of adipogenesis in liver and changes of insulin signaling

**Figure 5:**
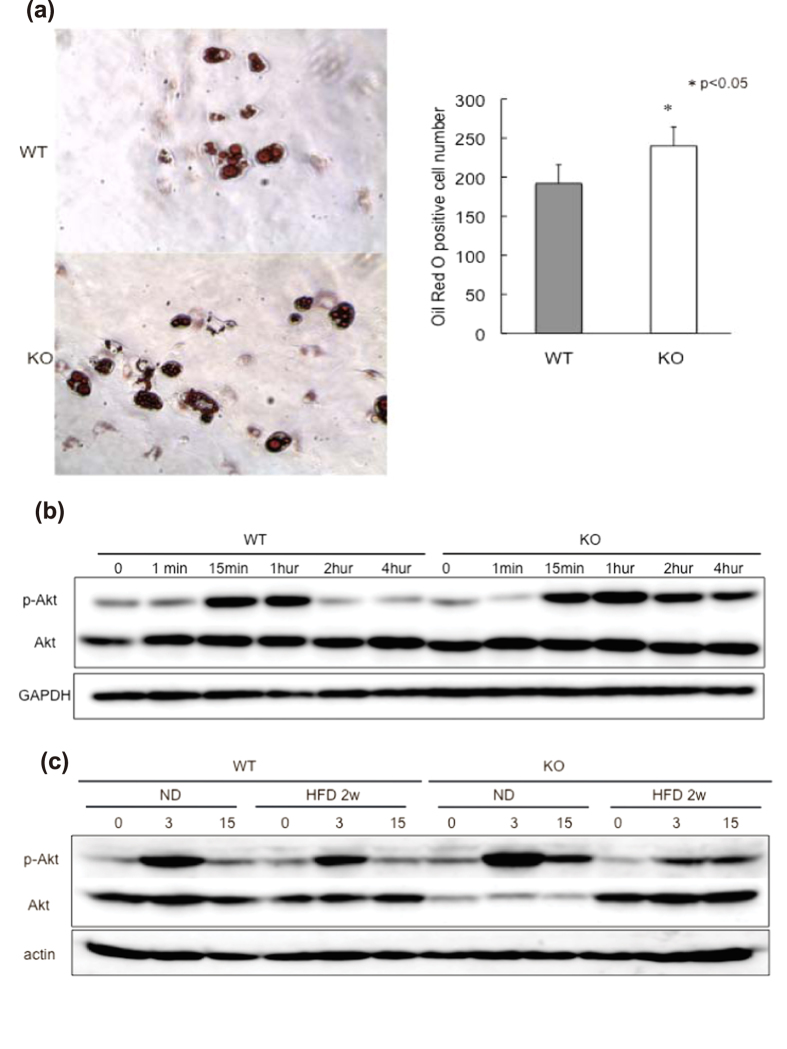
Conversion of insulin signaling in GADD34-deficient mice by aging and HFD feeding. (**a**) Addipocytes were proliferated and differentiated from WT and GADD34-deficient MEFs by insulin treatment. Picture shows the Oil Red O staining. The numbers of Oil red O positive cells shown are the mean ratio ± SEM. (Tree experiments *p < 0.05). (**b**) WT and GADD34-deficient MEFs were treated with insulin (10 μg/ml). Protein expression of p-Akt, Akt and GAPDH were analyzed by western blotting. The original immunoblots are presented in Supplemantary Figure 10c. (**c**) Three months old WT and GADD34-deficient mice were fed ND and HFD for 2 weeks. After 5 h starvation, insulin (5 U/mice) were injected intravenously. The livers were removed from these mice in each time (0 min, 3 min, 15 min). Protein expression of p-Akt, Akt and actin in the liver were analyzed by western blotting. The original immunoblots are presented in Supplemantary Figure 10d.

To determine the mechanism of HFD-induced fat accumulation in GADD34-deficient mice, it was necessary to examine the potential precursor cells that differentiate into fat cells. Mouse embryonic fibroblasts (MEFs) are frequently used to examine the adipocyte differentiation process^29^,^30^. Differentiation into fat is dependent on insulin signaling^31^,^32^. Here we examined MEFs *in vitro* as potential fat precursors and compared their capacity for adipocyte differentiation between WT and GADD34–deficient mice (Supplemental Fig. 7). MEFs cultured in insulin-containing medium were stained with Oil Red O. Higher numbers of adipocytes were observed in GADD34–deficient MEFs compared with WT MEFs (Fig. 5a). We examined insulin signaling in both WT and GADD34-deficient MEFs before differentiation. After insulin stimulation, GADD34-deficient MEFs showed higher levels of p-Akt compared with WT MEFs (Fig. 5b). The finding that insulin signaling is higher in GADD34-deficient MEFs than in WT MEFs contradicts the finding that GADD34-deficient mice showed lower levels of insulin signaling with age.

We hypothesized that higher level of insulin signaling in young male GADD34-deficient mice might be reduced with age. To test our hypothesis, we examined insulin signaling by injecting insulin into WT or GADD34-deficient mice before and after a HFD. We found that on ND, the expression of p-Akt was highly increased in the livers of young GADD34-deficient mice compared with WT mice. However, after 2 weeks of a HFD, the levels of p-Akt expression were actually decreased in GADD34-deficient mice relative to that in WT mice (Fig. 5c). We found almost the same results in fat and muscle tissues (Supplemental Fig. 5a). These results show that aging or a HFD resulted in lower insulin signaling in young GADD34-deficient mice compared with WT mice. We found that GADD34-deficient mice had a higher number of CD24^+^Sca-1^+^CD34^+^ cells in bone marrow than did WT mice after 2 weeks of a HFD (Supplemental Fig. 8).

**Figure 6:**
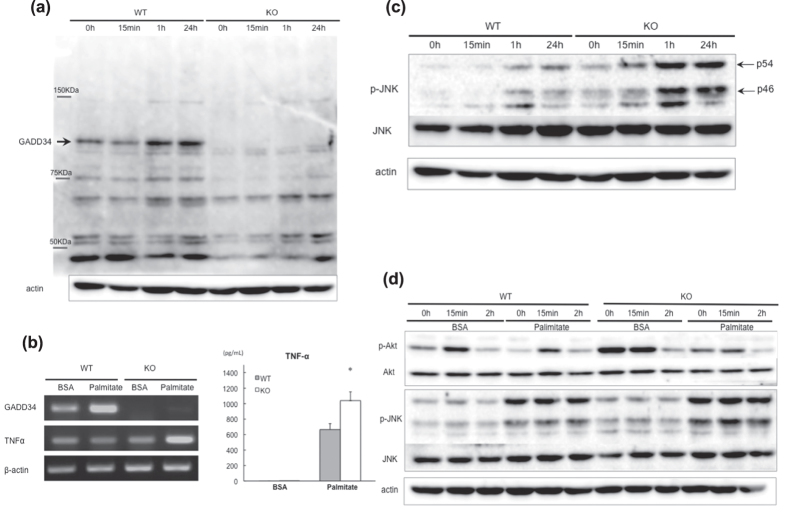
Palmitate induces phosphorylation of JNK and inhibits insulin signaling. WT and GADD34-deficient MEFs were treated with BSA (0.17 mM) or palmitate-BSA (0.2 mM/0.17 mM). (**a**) Protein expression of GADD34 were analyzed by western blotting. (**b**) Expression of TNF-α mRNA and production of TNF-α in MEF stimulated with BSA or BSA-palmitate. (**c**) Protein expression of p-JNK (JNK and β-actin as control) were analyzed by western blotting. The original immunoblots are presented in Supplemantary Figure 10d. (**d**) WT and GADD34-deficient MEFs were stimulated with insulin (10 μg/ml) after BSA (0.17 mM) or palmitate-BSA (0.2 mM/0.17M) treatment for 24 hours. Protein expression of p-Akt, p-JNK and β-actin at 0, 15 min or 2h were analyzed by western blotting. The original immunoblots are presented in Supplemantary Figure 10e.

To mimic the HFD condition, we stimulated MEFs with palmitate, which induced GADD34 expression in WT MEFs (Fig. 6a). Further, we found that palmitate induced higher levels of TNF-α expression, both at the mRNA and protein levels, in GADD34-deficient MEFs than in WT MEFs (Fig. 6b).

Concordant with TNF-α production, palmitate induced higher levels of p-JNK in GADD34-deficient MEFs than in WT MEFs (Fig. 6c). It has been shown previously that palmitate induces JNK activation via TLR signaling^33^. JNK phosphorylates insulin receptor substrates (IRS) 1 and 2 at serine or threonine residues and thereby attenuates their insulin-induced tyrosine phosphorylation, resulting in down-regulated insulin signaling and diminished AKT activation^34^. Indeed, the addition of palmitate reduced the insulin signaling stimulated by the addition of insulin in GADD34-deficient MEFs compared with WT MEFs (Fig. 6d).

Finally, we evaluated p-eIF2α expression in liver after HFD. Until two weeks p-eIF2α expressions were not significantly influenced by the HFD (Supplemental Fig. 9a). Also p-eIF2α expressions were not significantly influenced by aging (Supplemental Fig. 9b). Thus we suggest that dephosphorylation of p-eIF2α by GADD34 has only slight effects on obesity in HFD feeding. Life long changes of p-eIF2α expression were not so great. These results indicate that dephosphorylation of p-eIF2α by GADD34 was not the main cause of obesity, fatty liver and insulin resistance by aging.

Taken together, GADD34-deficient mice fed ND display up-regulated insulin-Akt signaling, which induces fat differentiation. This up-regulated insulin-Akt signaling is diminished by aging and a HFD, which ultimately proceeds to metabolic syndrome.

## Discussion

We found that GADD34 -deficient mice became obese followed by insulin resistance, fatty liver (NAFLD/NASH) and liver cirrhosis by advancing age. Further we have shown that liver of GADD34 -deficient mice accumulate fat droplets in hepatocytes and higher number of adipocytes by HFD. Previously Oyadomari *et al*.^35^, have shown that GADD34 transgenic mice attenuate eIF2α phosphorylation, which result in diminished hepatosteatosis. They showed that HFD induced eIF2α phosphorylation, which stimulated hepatic glycolysis by C/EBPα, C/EBPβ and PPARγ up-reguration following lipogenesis in WT mice. They showed that GADD34 overexpression dephosphorylated eIF2α phosphorylation and suppressed lipogenesis and glucogenesis. However, they did not show the data of eIF2α phosphorylation by HFD. When we examined the eIF2α phosphorylation by HFD, we found that HFD feeding did not clearly induce eIF2α phosphorylation in liver. Mechanism of steatosis in GADD34-deficient mice may be mainly comes from the effect of GADD34 on insulin signaling as discussed below, because eIF2α phosphorylation in GADD34-deficient mice was not significantly increased by HFD and aging (Supplementary Fig. 9a,b).

We have shown that induction of insulin signaling (p-Akt) by the stimulation of insulin in young GADD34-deficient mice was higher at normal condition than that in WT (Fig. 5c). Higher level of insulin signaling induced fat differentiation (Figs 2b and 5a), which induced obesity and steatosis in GADD34-deficient mice. However, induction of insulin signaling by the stimulation of insulin turned to be lower in GADD34-deficient mice than that in WT mice by HFD (Fig. 5c). Conversion of insulin signaling may come from two pathways. One is direct effect of HFD. The other is TNF-α production from infiltrated macrophages to fatty liver. Because HFD contains a large amount of fatty acids, we examined the effects of fatty acids included in HFD. We have shown here that palmitate activate JNK in MEFs. This activation was much higher in GADD34 deficient MEFs than that in WT MEFs. It has been shown that palmitate induces JNK activation via TLR signaling^33^. JNK phosphorylates insulin receptor substrates (IRS) 1 and 2 at serine (Ser) or threonine (Thr) residues and thereby attenuate their insulin-induced tyrosine (Tyr) phosphorylation, resulting in down-regulation of insulin signaling and diminished AKT activation^34^. Indeed we have shown that palmitate treatment activates JNK (Fig. 6c) and both palmitate and insulin treatment down-regulates p-Akt (down stream signaling of IRS1) in GADD34-deficient MEF (Fig. 6d).

Second cause of the conversion of insulin signaling comes from the cytokines from inflammatory cells. We have shown here that in aged GADD34 -deficient mice immune cells are recruited to the site of injury (portal area of the liver) (Fig. 3c), which generate pro-inflammatory cytokines, such as TNF-α, IL-6 and IL-1β (Fig. 1d). It has been shown that in NASH patients, portal macrophage accumulation is the earliest changes, and then follows T cells and B cells^36^. Obesity-associated tissue inflammation is now recognized as a major cause of insulin resistance^37^,^38^,^39^,^40^. TNF-α produced by obesity-induced inflammation stimulates IKK^41^, JNK^42^, and mTOR/S6K^43^,^44^, which enhance serine phosphorylation of insulin receptor substrate-1 (IRS-1). We also showed that IL-1β is enhanced in aged-GADD34 -deficient mice. It has been shown that HFD-induced fatty acids activate inflammasome, which releases IL-1β and induces insulin resistance^45^. IL-6 was also increased in aged GADD34 -deficient mice. Effects of IL-6 to insulin sensitivity are still controversial^46^.

These recruited monocytes have been shown to promote hepatic fibrosis^47^. Then followed the production of TGF-β, which stimulate hepatic stellate cells, portal fibroblasts, and myofibroblasts of bone marrow origin and induce to produce collagen^48^. Hepatic stellate cells become activated by liver injury (here HFD-caused liver injury) and trans differentiate to myofibroblasts-like cells^49^. We detected α-SMA positive fibroblast around portal area (Fig. 3c, Supplementary Fig. 1a, 6g). These cells may cause liver fibrosis. Hepatic fibrosis is the wound-healing response of the liver to chronic injury. Here, NASH is the cause of hepatic injury, which induces hepatic damage by inflammation. Matrix deposition such as type I collagen, parenchymal cell death angiogenesis leads to progressive fibrosis^49^.

Interesting points, we observed here, were conversion of insulin signaling, before and after HFD treatment. This conversion was observed both *in vivo* injection of insulin and *in vitro* treatment of MEFs by insulin with or without palmitate treatment. GADD34 -deficient mice had higher level of insulin signaling before HFD, which correlate the higher level of insulin signaling of MEFs without palmitate treatment. After HFD or with palmitate treatment GADD34 -deficient mice showed insulin resistance. Insulin signaling of MEFs without palmitate treatment has two meaning. Although MEFs has a capacity to differentiate to adipose tissue, MEFs also work as one of a tissue of glucose metabolism. Our results presented here by MEFs correlate to age-related insulin resistance. Young GADD34 -deficient mice are higher level of insulin signaling, which changed to insulin resistance by aging. These conversions may be caused by higher induction of TNF-α followed by the higher induction of p-JNK in GADD34 -deficient mice, which has been shown to be main pathway to insulin resistance.

We showed that insulin signaling in young GADD34 -deficient mice was higher than that of WT mice, however, the GTTs and ITTs were comparable between GADD34-deficient and WT mice in their younger age (Fig. 1b). In order to explain these results, we measured insulin levels in normal diet, HFD for two weeks and aging. After 5 h starvation (fasted), insulin levels were not different between young (2.5M) GADD34 -deficient and WT mice. We injected glucose 2 g/kg after fasted 5 h and measured serum insulin levels after 30 min of glucose injection (fed). We found that insulin levels were lower at 30 min in young GADD34 -deficient mice than those in WT mice (Supplement Fig. 4a). These results correlate to the higher insulin signaling in young GADD34 -deficient mice. After 2 weeks of HFD, insulin levels of fasted (5 h starvation) and glucose injection (fed) were higher in GADD34 -deficient mice that those in WT mice (Supplement Fig. 4b). In aged (15M) mice, insulin level of fasted was higher in GADD34-deficient mice than those in WT mice (Supplement Fig. 4c). In aged mice, insulin levels measured at 30 min after glucose injection were slightly higher in GADD34-deficient than those in WT mice (Supplement Fig. 4d). These results correlate to the finding that insulin signaling is higher in young GADD34-deficient than WT mice. Thus young GADD34-deficient mice do not need to high level of insulin secretion by glucose injection.

Taken together it will be concluded that GADD34 -deficient mice got higher fat in liver by aging and HFD. GADD34 suppress adipocyte differentiation by decreasing insulin-Akt signaling. GADD34 -deficient mice become insulin resistance by aging, which is caused by the decrease of insulin-Akt signaling.

## Materials and Methods

### Mice

Two-month-old C57BL/6 (C57BL/6N; B6) mice were purchased from SLC Japan. The GADD34 gene was derived originally from the 129 mouse strain. The neo gene was inserted inversely into the GADD34 gene after the first Met residue and then introduced into ES cells derived from strain 129 by homologous recombination. ES cells were injected into 129 × B6 mouse blastocysts to establish chimeras. The germ line-transmitted chimeric mice were obtained and established as GADD34-deficient mice^13^. These mice were then mated with the B6 strain and back-crossed for 10 generations. We used GADD34-deficient mice with the C57BL/6 background. These mice were maintained on a normal diet (ND; CLEA Rodent Diet CE-2) or high-fat diet (HFD; CLEA High Fat Diet 32) under 12-hour light and dark cycles and specific pathogen-free conditions in the Animal Research Facility at the Nagoya University Graduate School of Medicine and were used according to institutional guidelines. All studies were approved by the Animal Care and Use Committee of Nagoya University Graduate School of Medicine approved the protocol.

### Biochmical analysis

Serum analysis of liver marker enzymes ALT, AST, TG and Cholesterol, which are fat mediators, were measured by WAKO assay kits (Wako Pure Chemicals, Osaka, Japan). Glycogen measured it by method from Bio-protocol^50^. Insulin in serum was measured by Mouse insulin ELISA KIT (TMB)(AKRIN-011T, Shibayagi, Gunma, Japan). IL-6, TNF-α and IL-1β in serum from WT mice and GADD34 -deficient mice or TNF-α in culture supernatant from WT mice and GADD34 -deficient MEFs were measured by the ELISA Kit (R&D Systems, Wiesbaden, Germany).

### Glucose and insulin tolerance test

For the glucose tolerance test, WT and GADD34-deficient mice aged 3 and 15 months were fasted 5 hours and injected i.p. with a glucose solution (1.5 g/kg). For the insulin tolerance test, mice were fasted for 5 hours and injected i.p. with bovine insulin (0.75 mU/kg, Sigma-Aldrich, St. Louis, MO, USA). Blood samples were collected from the tail at each time point, and blood glucose was measured using the LaboAssay^TM^ Glucose kit (Wako Pure Chemicals, Osaka, Japan).

### Histological analysis

Liver tissue was fixed in 4% formaldehyde buffered with PBS (pH 7.4) and blocked in paraffin. Paraffin-embedded 4-μm sections were stained with hematoxylin for 5 minutes and with eosin for 3 minutes. For Masson Trichrome staining, paraffin-embedded sections were fixed in Bouin’s Solution and then washed with distilled water and stained with Weigert’s iron hematoxylin solution for 10 minutes. After washing with distilled water, the sections were stained with Biebrich Scarlet for 5 minutes. For detection of collagen, sections were incubated with phosphotungstic/phosphomolybdic acid solution for 10 minutes and then with aniline blue for 5 minutes. Finally, the stain was fixed with 1% acetic acid for 1 minute.

After embedding with O.C.T. compound (Sakura Finetechnical Co., Ltd., Tokyo, Japan), frozen 5-μm liver sections were stained for immunohistochemical analyses to evaluate immune cells using anti-GR-1-FITC, CD11b-PE, CD11c-PE, c-Kit-FITC (all from BD Biosciences, San Jose, CA, USA), CD4-FITC, CD8-PE, F4/80-APC (all from eBioscience, San Diego, CA, USA) and α-SMA-Cy3 (Sigma-Aldrich) antibodies. For hepatoma detection, liver sections were stained with anti-α-fetoprotein (Santa Cruz Biotechnology, Inc., Santa Cruz, CA, USA) and then donkey anti-goat IgG-FITC (Santa Cruz Biotechnology, Inc.) as a second antibody. Other sections were stained with Oil Red O for fatty liver analysis. Frozen sections were fixed in 10% formaldehyde, washed with distilled water, rinsed with 60% isopropanol, stained with Oil Red O (Wako Pure Chemicals, Osaka, Japan) working solution (in 0.18% isopropanol) for 20 minutes, rinsed with 60% isopropanol and stained with hematoxylin for detection of the nuclei.

### Flow cytometry analysis

Cells were stained with several fluorescently labeled anti-mouse antibodies as follows. Adipocyte precursors were identified using CD34-PE, CD24-FITC, and Sca-1-APC antibodies. Immune cell infiltration into the epididymal WAT was detected using Gr1-APC, CD11b-PE, Ly6G-PE, F4/80-APC, CD11c-FITC and Ly6C-APC. Cells were analyzed using the FACSCanto flow cytometer (BD Biosciences). Data were analyzed using FlowJo software (TreeStar Inc., Ashland, OR, USA).

### Western blotting

Tissues were homogenized in buffer (50 mM Tris-HCl pH 6.8, 2% SDS, 2 mM EDTA-2Na) and then lysed in sample buffer (50 mM Tris-HCl pH 6.8, 2% SDS, 2.5% glycerol, 5% 2-mercaptoethanol, and 0.05% bromophenol blue). Total protein (1 mg/mL) was resolved by SDS-PAGE and then transferred to PVDF membranes (Millipore, Billerica, CA, USA).

Cells (3 × 10^5^/dish) were lysed in sample buffer (62.5 mM Tris–HCl, pH 6.8, 2% SDS, 10% glycerol, 5% 2-mercaptoethanol, and 5% bromophenol blue). Protein concentrations were quantified by Bradford assay using the Bio-Rad protein assay kit (Bio-Rad Laboratories, Inc., Hercules, CA, USA). Total protein (50 μg/lane) was resolved by SDS-PAGE and transferred to PVDF membranes (Millipore).

The membranes were reacted with anti-phospho Akt (Akt2), anti-Akt (Akt2), anti-PPARγ, anti-GAPDH (Cell Signaling, Danvers, MA, USA) or anti-GADD34 (sc-825; Santa Cruz Biotechnology) overnight at 4 °C and then with anti-rabbit IgG HRP-conjugated secondary antibody (GE Healthcare, UK Ltd). Signals were detected by enhanced chemiluminescence (ECL) system (GE Healthcare, UK Ltd).

### RT-PCR analysis

Total RNA was isolated using TRIzol reagent (Invitrogen, Carlsbad, CA, USA) according to the manufacturer’s recommended protocol. Residual genomic DNA was digested and removed using DNase I (Invitrogen) treatment. First-strand cDNA was synthesized using 1 μg total RNA and the High Capacity cDNA Reverse Transcription Kit (Applied Biosystems). Quantitative real-time PCR analysis was performed using the MX3000P QPCR System (Agilent Technologies, Santa Clara, CA, USA) and SYBR Green Realtime PCR Master Mix (TOYOBO, Osaka, Japan) according to the manufacturer’s instructions. The following program was used: ten sec. at 95 °C, followed by 40 cycles of 5 sec at 95 °C and 20 sec at 58 °C. The following primers were used:

β-actin, (f) 5′-AGTGTGACGTTGACATCCGT-3′ and

(r) 5′-GCAGCTCAGTAACAGTCCGC-3′;

GADD34, (f) 5′-CTTTTGGCAACCAGAACCG-3′ and

(r) 5′-CAGAGCCGCAGCTTCTATCT-3′;

PPARγ, (f) 5′- ACATAAAGTCCTTCCCGCTGACCA-3′ and

(r) 5′- AAATTCGGATGGCCACCTCTTTGC-3′.

TNF-α, (f) 5-′GCCCATATACCTGGGAGGAG-3′ and

(f) 5-′CACCCATTCCCTTCACAGAG -3′

Scd1, (f) 5-′CAGCCTGTTCGTTAGCACCTTCTTG-3′ and

(f) 5-′CACTGGCAGAGTAGTCGAAGGGGAAG -3′

Srebf1, (f) 5-′CTGCCCGGACACACCAGCTC-3′ and

(f) 5-′TGCCCAGGAGCCGACAGGAA -3′

Values were normalized to β-actin mRNA expression levels.

### Mouse embryonic fibroblast adipogenesis assay

MEFs were prepared from 14.5-day post-coital mouse embryos and digested with trypsin. They were cultured in Dulbecco’s Modified Eagle’s Medium (DMEM) supplemented with 10% fetal bovine serum (FBS), 50 U/ml penicillin and 50 mg/ml streptomycin. Confluent MEFs from WT and GADD34-deficient mice were used for adipogenic differentiation assays by incubating first with 10 μg/ml insulin, 250 nM dexamethasone and 0.5 mM isobutylmethylxanthine (Sigma-Aldrich) for 2 days, and then 10 μg/ml insulin was added every 2 days. After 10 days, we analyzed adipocyte differentiation by Oil Red O staining.

### Palmitate stimulation

MEFs from WT and GADD34-deficient mice were plated at 3 × 10^5^ cells/well in 6-well dishes and treated with 0.17 mM bovine serum albumin (BSA) or 1 mM palmitate/0.17 mM BSA (6:1 ratio palmitate:BSA). Palmitate/BSA conjugates were prepared by mixing sodium palmitate (Sigma-Aldrich) with Hsp protease FFA-free BSA (Sigma-Aldrich) according to the protocol from Seahorse Bioscience Inc. (North Billerica, MA, USA).

### Statistical analyses

Data are expressed as means ± standard error of the mean (SEM). Statistical comparisons were performed using ANOVA, followed by Fisher’s post hoc test. p-values < 0.05 were considered statistically significant.

## Additional Information

**How to cite this article**: Nishio, N. and Isobe, K.-I. GADD34-deficient mice develop obesity, nonalcoholic fatty liver disease, hepatic carcinoma and insulin resistance. *Sci. Rep*. **5**, 13519; doi: 10.1038/srep13519 (2015).

## Supplementary Material

Supplementary Information
