# Supplementary material for: GADD34-deficient mice develop obesity, nonalcoholic fatty liver disease, hepatic carcinoma and insulin resistance
Source: Sci Rep. 2015 Aug 28;5:13519. doi: 10.1038/srep13519 (PMC4551985; doi:10.1038/srep13519)

Supplementary Information

GADD34-deficient mice develop obesity, nonalcoholic fatty liver disease, hepatic carcinoma and insulin resistance

Naomi Nishio and Ken-ichi Isobe

Department of Immunology, Nagoya University Graduate School of Medicine, 65 Turumai-cho, Showa-ku, Nagoya, Aichi, 466-8550, Japan

Correspondence: K. Isobe, Department of Immunology, Nagoya University Graduate School of Medicine, 65 Tsurumai-cho, Showa-ku, Nagoya, Aichi, 466-8550, Japan.

Email address: kisobe@med.nagoya-u.ac.jp

Tel: +81-52-744-2135

Fax: +81-52-744-2972

Supplemental Figure 1.

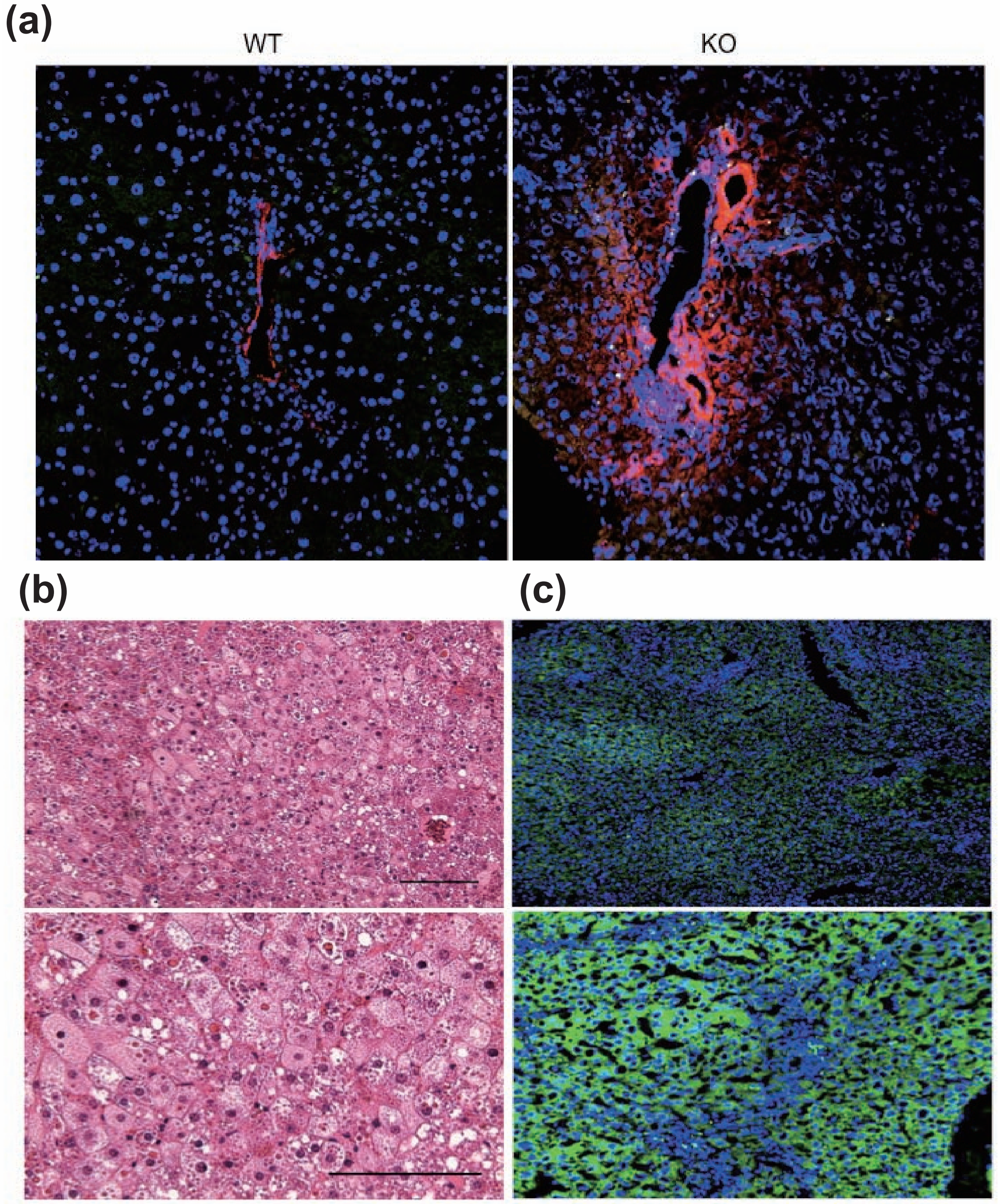

Supplemental Figure 1; GADD34 deficiency induced the liver diseases in aged mice.

(a) Frozen sections of liver from aged WT and GADD34 -deficient mice were stained with anti-αSMA-Cy3 (Red). Analyzed by confocal microscopy.

(b) H& E section of Hepatocellular carcinoma from the liver of male GADD34 -deficient 17 M mice.

(c) Immunohistological staining by FITC-labeled anti-AFP (Green) was analyzed by confocal microscopy.

Supplemental Figure 2

(a)

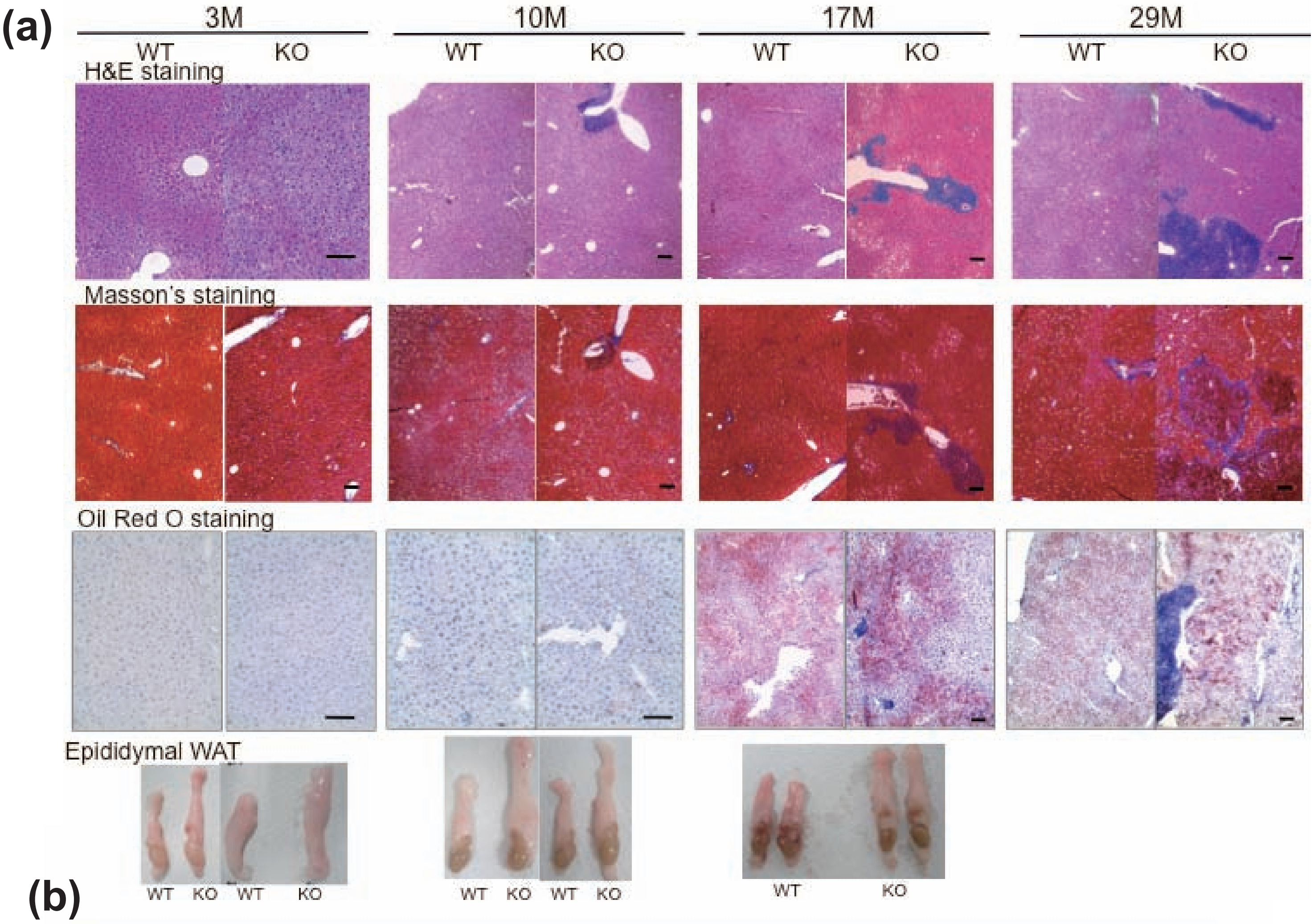

(b)

	3M	5M	6M	8M	10M	12M	14M	15M	16M	17M	18M	20M	20M～
WT	0/100	0/4	0/4	0/2	0/4	0/2	0/1	0/0	0/2	0/4	0/2	0/2	0/7
KO	0/100	0/4	4/10	1/2	5/9	2/4	2/6	2/3	4/6	4/4	2/3	4/4	8/12

	WT	KO
3M	0/100	0/100
5-7M	0/8	4/14
8-10M	0/6	7/11
12-20M	0/14	20/30
20M～	0/7	8/12

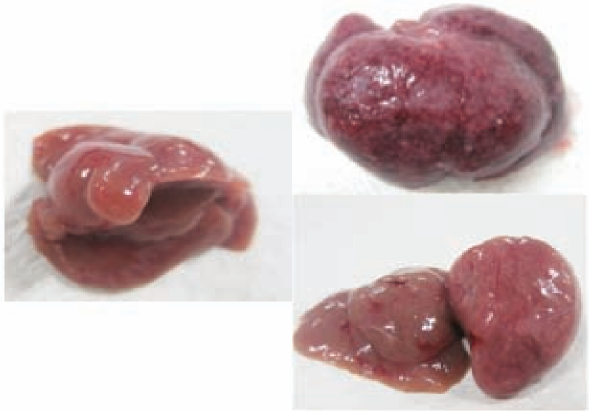

Supplemental Figure 2; GADD34 deficiency induced the liver diseases in aged mice.

(a) Sections of liver from male WT and GADD34 -deficient mice at different age were stained with H&E, Masson' s Trichrome and Oil Red O. Scale bars represent 100 μm. Photographs of Epididymal WAT of each age were shown below. The pictures show the male WT mice (left) and GADD34 -deficient mice (right) in each age.

(b) Data shows liver diseases (liver cirrhosis and liver carcinoma) / analyzed mice. The pictures show the representative macroscopic photographs of livers from aged GADD34-deficient mice.

Supplementary Figure 3

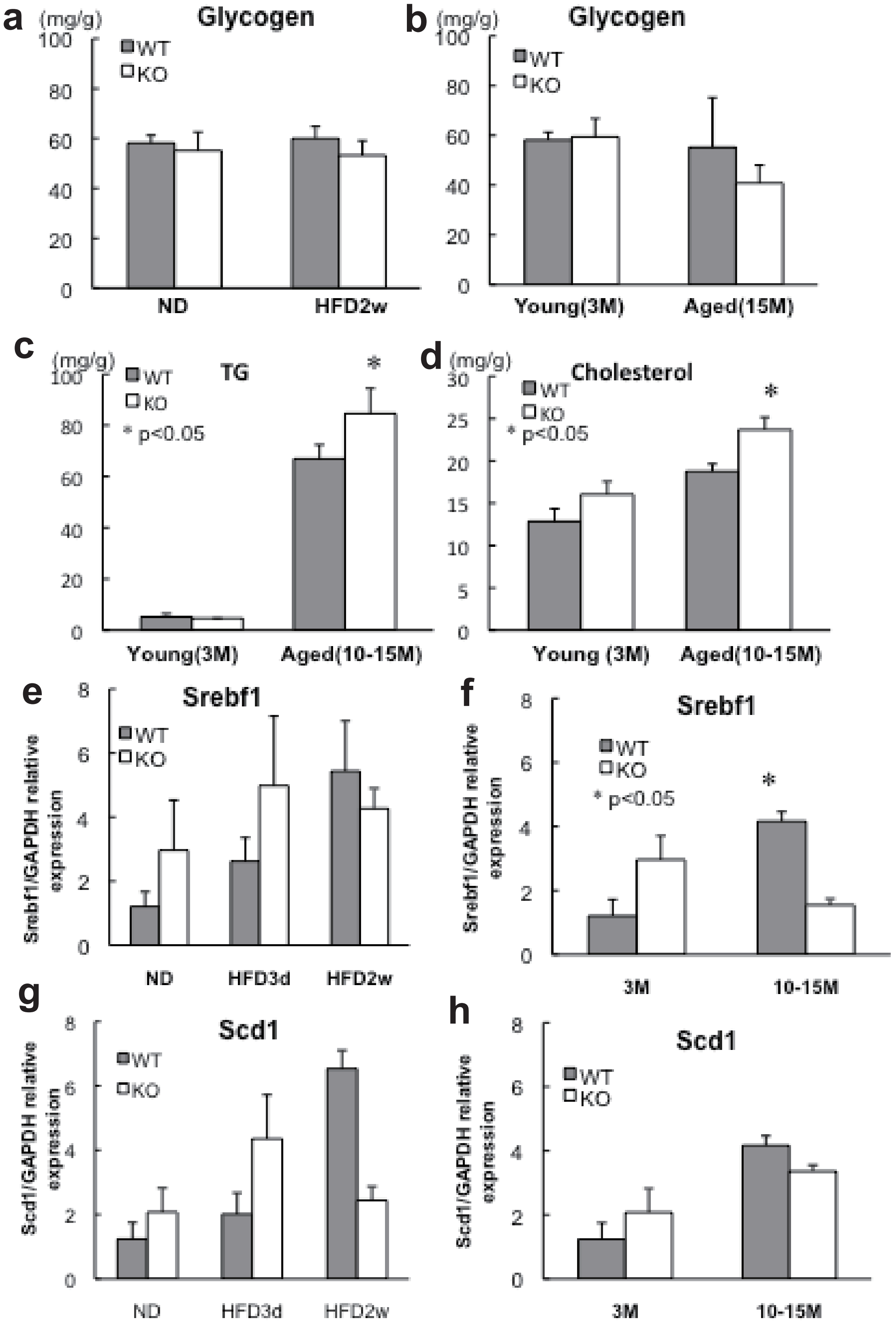

Supplementary Figure 3; Hepatic glycogen, TG and Cholesterol contents in erWT and GADD34 KO. Differences of hepatic glycogen contents between normal diet (ND) and HFD.

Liver tissues were taken from the 2.5M old WT and GADD34-deficient mice fed on a normal diet (ND) or the mice after 2 weeks of HFD (a). Data shown are the mean ratio ± SEM of three mice. (b-d) Differences of hepatic glycogen (b), TG (c) or cholestrrol (d) contents between young and aged mice. Liver tissues were taken from the 3M old or 10-15M old GADD34-deficient and WT mice fed on a ND or the mice after 2 weeks of HFD. RNAs were taken from the liver of control, at 3 days and at 2 weeks of HFD (e, g) or young (3M) and aged (10-15 M) (f, h) GADD34 -deficient or WT mice. Expressions of Srebf1, Scd1 mRNA were measured by Real time PCR analysis. Data shown are the mean ratio ± SEM of four mice.

Supplemental Figure 4

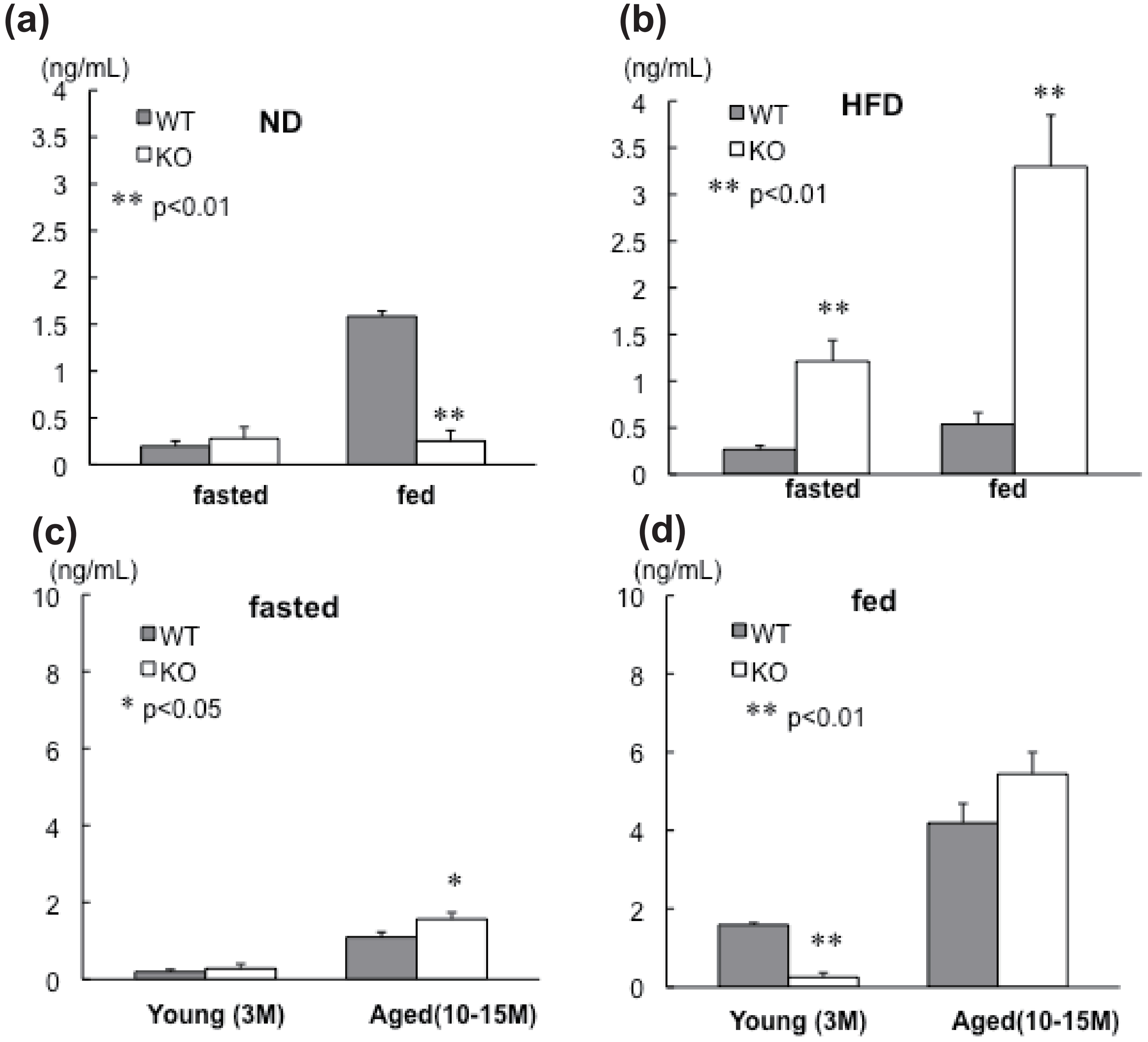

Supplemental Figure 4; Serum insulin levels of normal diet, HFD and aging in GADD34 KO and WT mice.

Serum insulin levels were measured in normal diet (a), at 2 weeks HFD (b) and aging (c) of WT and GADD34-deficient mice. After 5 h starvation, serum insulin levels were measured by taking serum (fasted) or taking serum after 30 min. of intraperitoneal glucose (2g/kg) injection in young (2.5M) GADD34-deficient and WT mice (a). Same experiments were done in the mice of two weeks of HFD (b), and young (3M) and aged (15M) mice with normal diet (c). Data shown are the mean ratio ± SEM of four mice.

Supplemental Figure 5

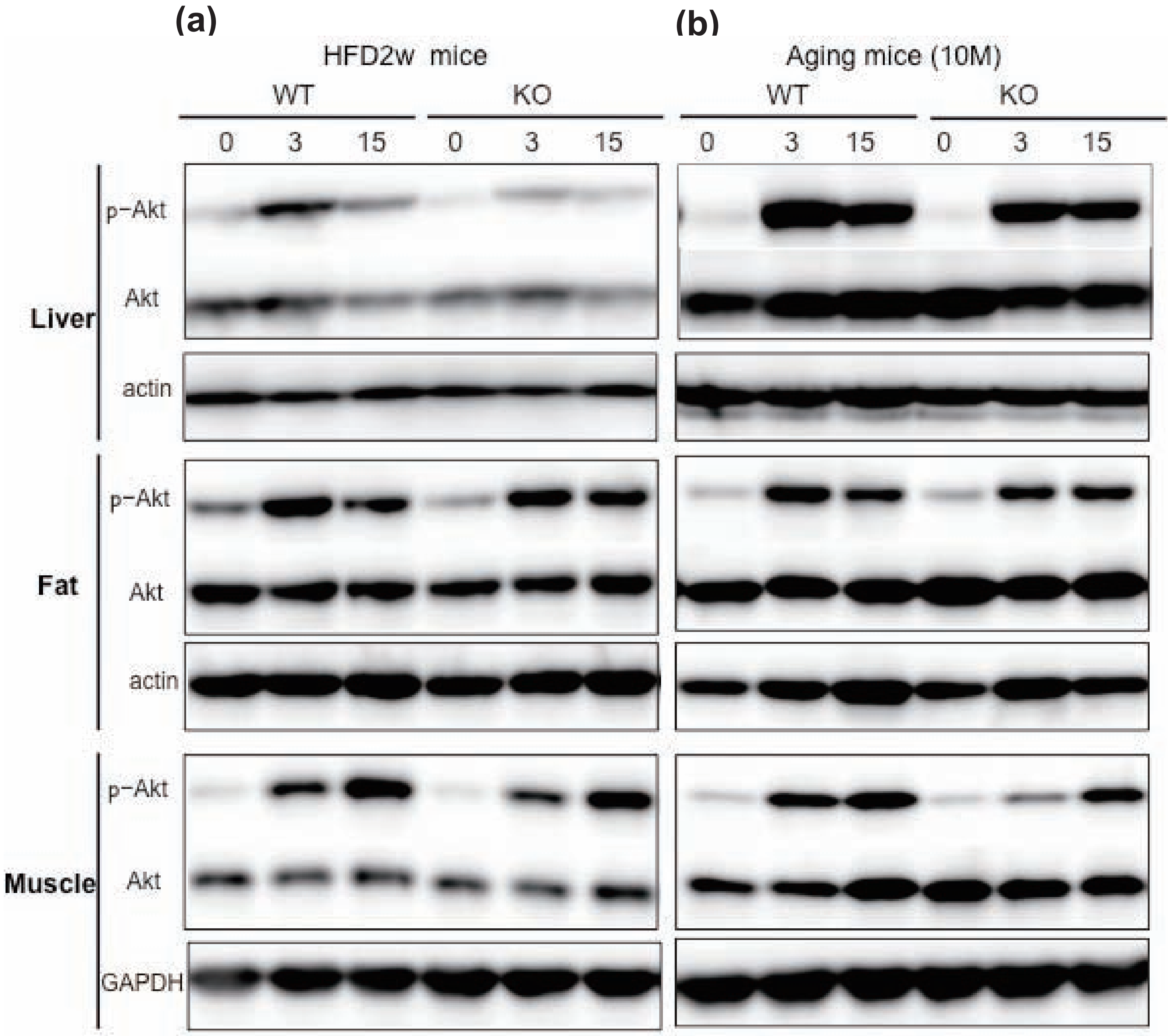

Supplemental Figure 5; GADD34 deficiency induced the insulin resistance in each tissue

WT and GADD34-deficient mice fed HFD for 2 weeks (a) and young (3M) and aged (10M) with normal diet (b) were fasted for 5 h then were injected insulin (5U/mice) to intravenously under anesthesia and the liver, fat and muscle were removed from these mice in each time (0 min, 3min, 15min). Protein expressions of p-Akt, Akt and GAPDH in liver were analyzed by western blotting.

Supplemental Figure 6

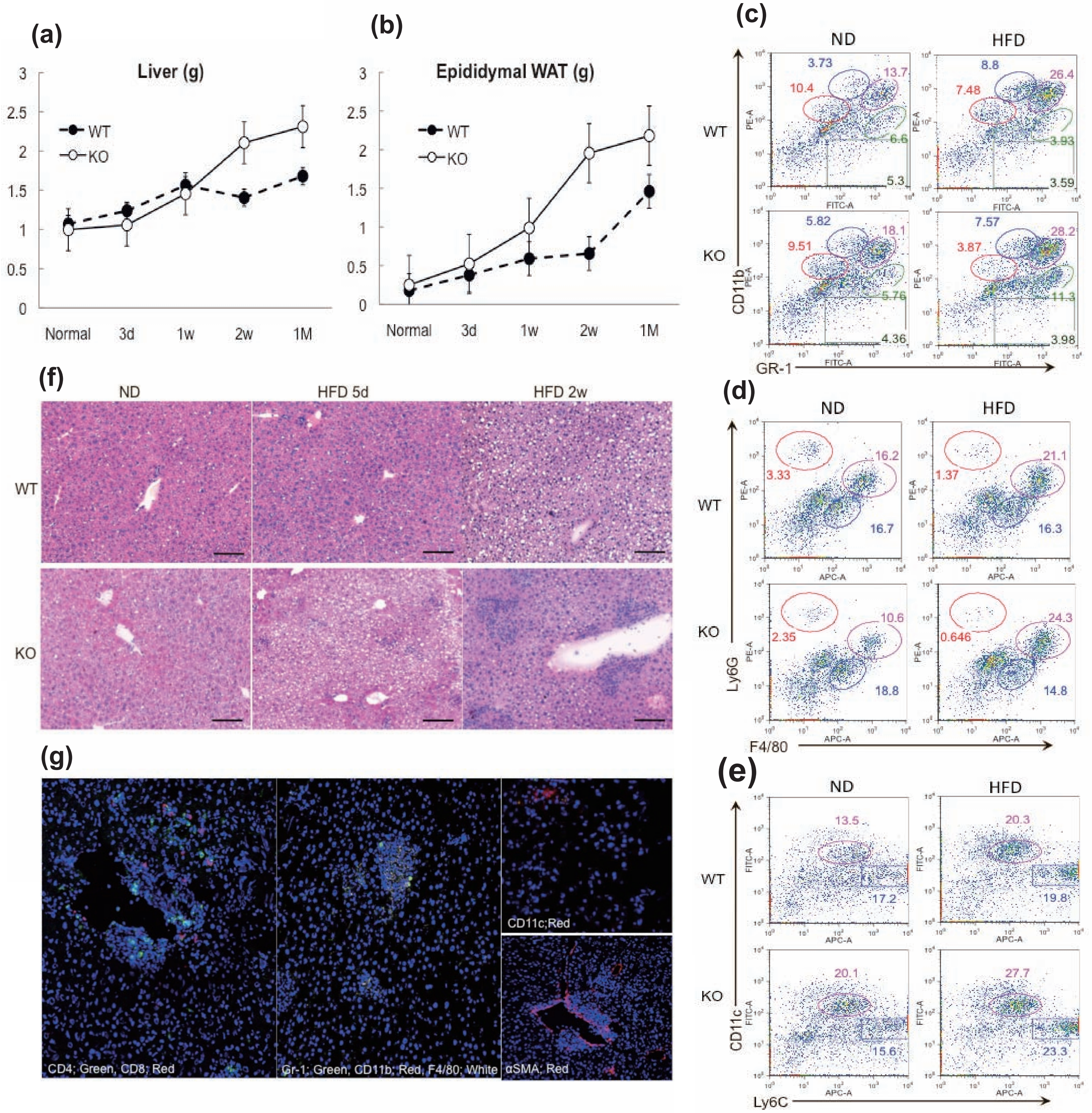

Supplemental Figure 6; Higher number of myelid lineage cells migration to epididimal WAT of GADD34 deficient mice by HFD, Related to Figure 4

(a) Liver (b) Epididimal WAT weight of 3 M old male WT and GADD34-deficient mice. (c-e) Epididimal WAT taken from 3 M old male WT and GADD34-deficient mice fed on a ND or 2 weeks of HFD were analyzed by FACS. Immune cells from WAT were stained with anti-GR-1-APC/anti-C11b-PE (c), anti-F4/80-APC/ anti-Ly6G-PE (d) or anti-Ly6c-APC/ anti-C11c-FITC (e). (f-g) Liver was extracted from 3 months old female WT and GADD34-deficient mice at 5 days and 2 weeks after HFD or ND. (f) Sections were stained with H&E. Scale bars represent 100 μm. (g) Frozen sections of liver from GADD34 -deficient mice were stained with anti-CD4-FITC (Green)/anti-CD8-PE (Red), anti-GR-1-FITC(Green)/anti-CD11b-PE (Red)/anti-F4/80-APC (white), anti-CD11c-PE (Red) and anti-αSMA-Cy3 (Red).

Suplemental Figure 7

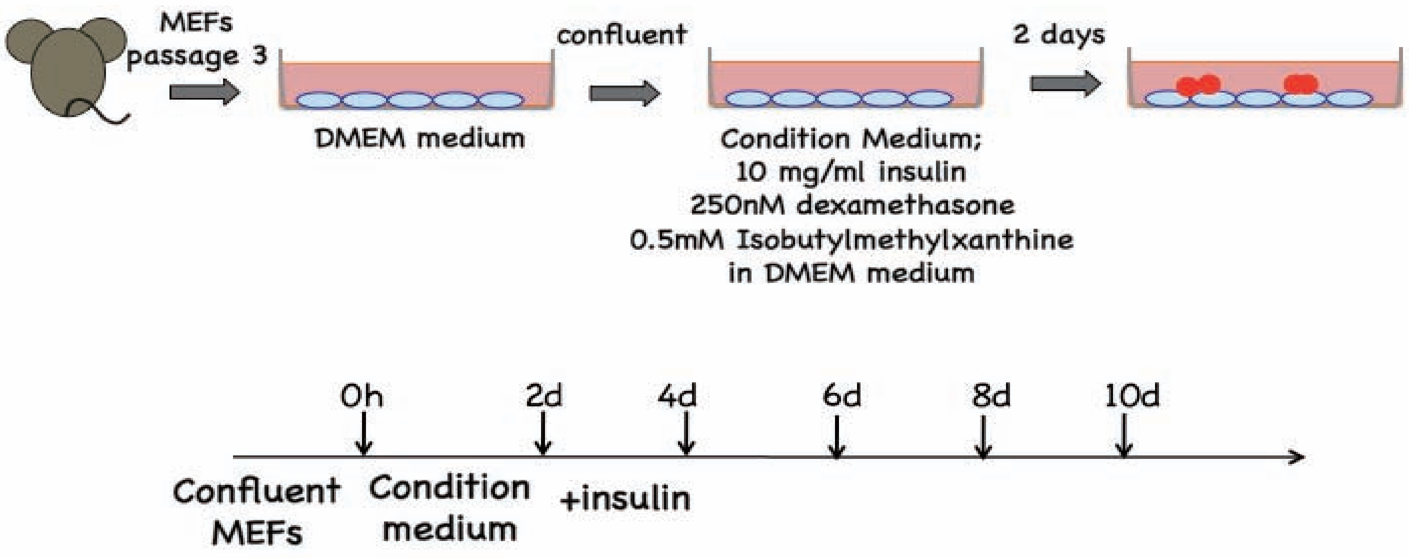

Supplemental Figure 7; cematic Protocol of Mouse embryonic fibroblast adipogenesis assay.

MEFs were prepared from 14.5-day post-coital mouse embryos and digested with trypsin.

They were cultured in Dulbecco' s Modified Eagle' s Medium (DMEM) supplemented with 10% fetal bovine serum (FBS), 50 U/ml penicillin and 50 mg/ml streptomycin. Confluent MEFs from WT and GADD34-deficient mice were used for adipogenic differentiation assays by incubating first with 10 μg/ml insulin,

250 nM dexamethasone and 0.5 mM isobutylmethylxanthine (Sigma-Aldrich) for 2 days, and then 10 μg/ml insulin was added every 2 days.

Supplemental Figure 8

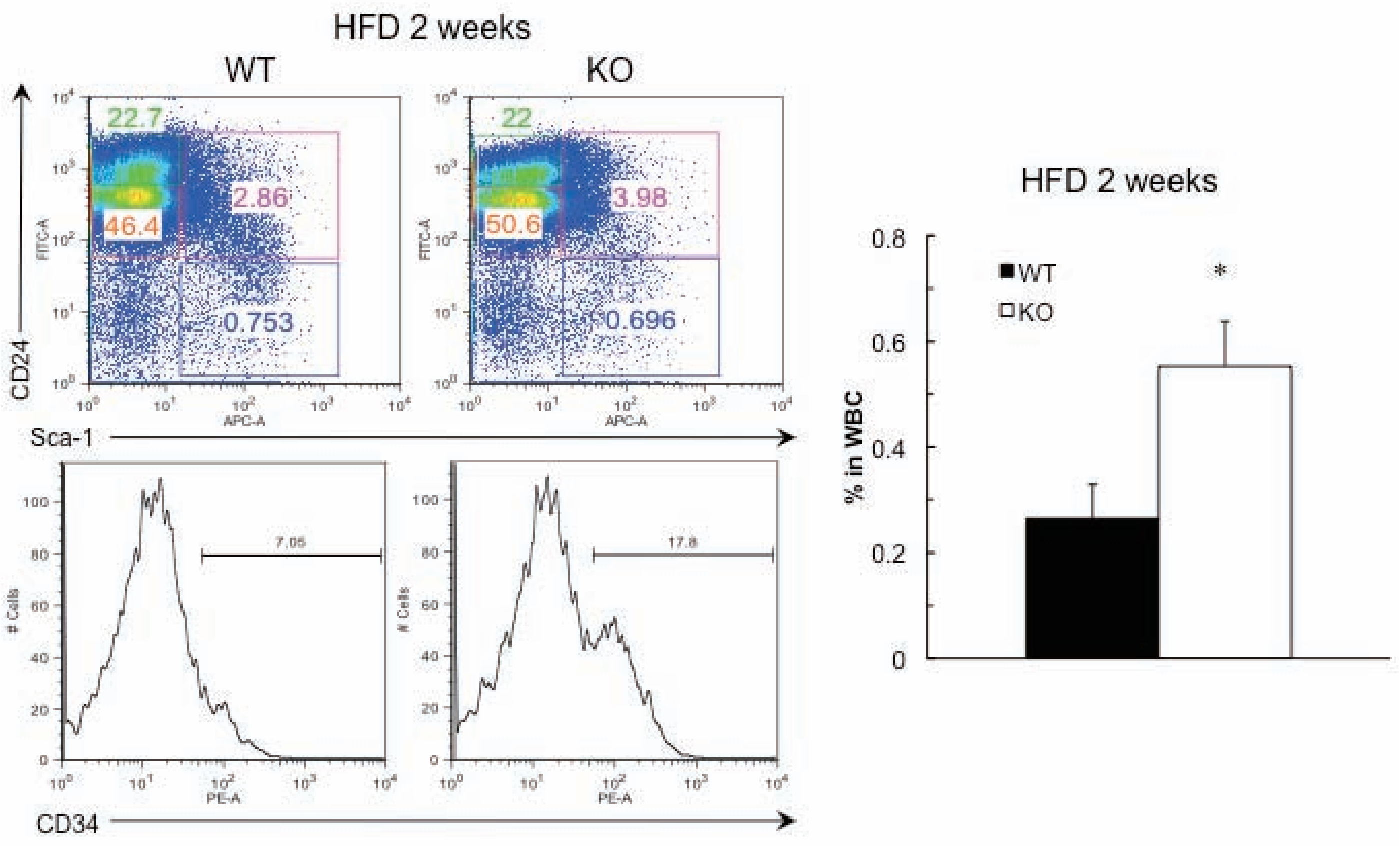

Supplemental Figure 8; Adipocytes progenitor cells were increased in GADD34-deficient mice by HFD.

FACS analysis of bone marrow cells from WT and GADD34 -deficient mice fed on a HFD.

Bone marrow cells were stained with anti-CD24, Sca-1 and CD34 antibody. Graph shows CD24+/Sca-1+/CD34+ adipocyte progenitor bone marrow cells. Representative results of three independent experiments are shown. Data shown are the mean ratio ± SEM. (*; $p<0.05$)

Supplemental Figure 9

a

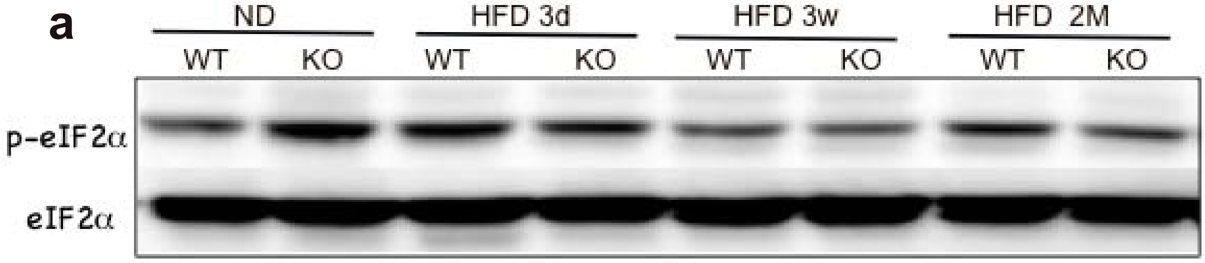

b

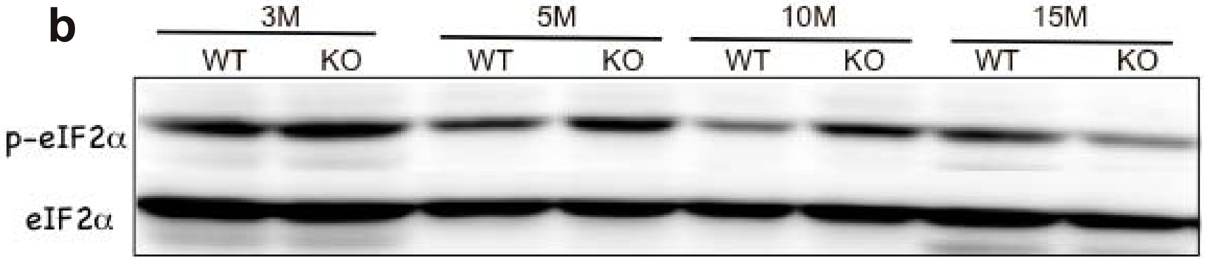

Supplemental Figure 9; Expression of p-eIF2α by HFD feeding and aging.

(a) Expression of p-eIF2α and eIF2α were analyzed by western blotting in the liver taken from different days of WT and GADD34-deficient mice fed on a ND or HFD.

(b) Expression of p-eIF2α and eIF2α were analyzed by western blotting in the liver taken from different ages of WT and GADD34-deficient mice fed on a ND.

Supplemental Figure 10

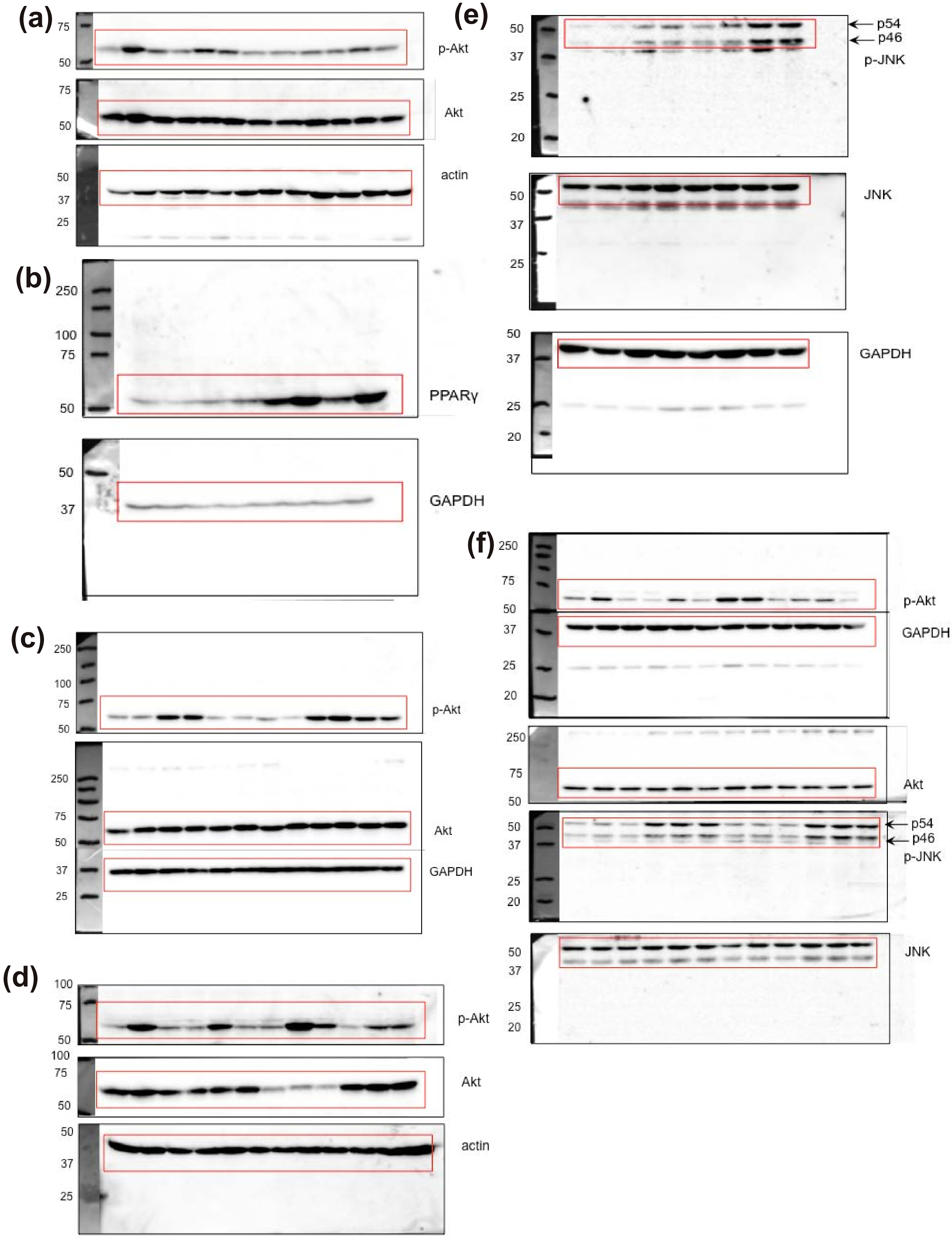

Supplement: Supplementary Information [file srep13519-s1.pdf]
